# Therapeutic implications of homologous repair deficiency testing in patients with prostate cancer (Part 2 of 2)

**DOI:** 10.1038/s41391-024-00887-z

**Published:** 2024-09-27

**Authors:** Anthony V. Serritella, Amy Taylor, Michael C. Haffner, Wassim Abida, Alan Bryce, Lawrence I. Karsh, Scott T. Tagawa, Przemyslaw Twardowski, Andrew J. Armstrong, Joshua M. Lang

**Affiliations:** 1 University of Wisconsin, Madison, WI USA; 2 Fred Hutchinson Cancer Center, Seattle, WA USA; 3 Memorial Sloan Kettering Cancer Center, New York, NY USA; 4 Mayo Clinic Arizona, Phoenix, AZ USA; 5 The Colorado Urology Center, Denver, CO USA; 6 Weill Cornell Medicine, New York, NY USA; 7 Saint John’s Cancer Institute, Santa Monica, CA USA; 8 Duke Cancer Institute Center for Prostate and Urologic Cancers, Duke University Medical Center, Durham, NC USA

**Keywords:** Cancer therapy, Cancer genetics

## Abstract

**Background/Objectives:**

Unfortunately, not all metastatic castration-resistant prostate cancer (mCRPC) patients receive available life-prolonging systemic therapies, emphasizing the need to optimize mCRPC treatment selections. Better guidelines are necessary to determine genetic testing for prostate cancer.

**Subjects/Methods:**

In this two-part expert opinion-based guide, we provide an expert consensus opinion on the utilization of germline and somatic testing to detect HRR alterations in patients with mCRPC. This guide was developed by a multidisciplinary expert panel that convened in 2023-2024, including representatives from medical oncology, urology, radiation oncology, pathology, medical genomics, and basic science.

**Results/Conclusions:**

In this second part, we highlight how genetic testing can lead to improved, life-prolonging mCRPC therapeutic strategies based on a review of the recent phase III trials and subsequent regulatory approvals for PARP inhibitors in mCRPC.

## Introduction

Poly(ADP-ribose) polymerase (PARP) inhibitors are life prolonging for patients with metastatic castration-resistant prostate cancer (mCRPC) harboring homologous recombination repair (HRR) gene alterations [1–6]. PARP is a DNA repair enzyme involved in the repair of single-strand breaks. When PARP is inhibited, single-stranded-DNA-breaks accumulate, leading to the accumulation of double-strand breaks (DSBs). A set of repair mechanisms are then activated, including homologous recombination repair (HRR). DSB repair relies primarily on HRR. Hence, aberrations within the HRR pathway, particularly germline or somatic deleterious mutations in *Breast cancer gene-1 (BRCA1) BRCA1* or *Breast cancer gene-2(BRCA2)*, can result in synthetic lethality in the presence of PARP inhibitors [7].

In recent years, PARP inhibitors have been combined with androgen receptor signaling inhibitors (ARSIs) in frontline mCRPC therapy with clear benefits for a subset of patients identified via genomic profiling. To identify those patients who are most likely to benefit, somatic and germline testing has been recommended as a standard of care by many society guidelines, including the National Comprehensive Cancer Network (NCCN) and American Urological Association (AUA) [8–10].

In this two-part expert opinion-based guide, we provide an expert consensus opinion on the utilization of germline and somatic testing to detect HRR alterations in patients with mCRPC. This guide was developed by a multidisciplinary expert panel that convened in 2023–2024, including representatives from medical oncology, urology, radiation oncology, pathology, medical genomics, and basic science. We argue for the widespread adoption of germline testing for all patients at the time of prostate cancer (PCa) diagnosis. Patients with metastatic hormone-sensitive PCa (mHSPC) or mCRPC would subsequently qualify for somatic tumor genetic testing to determine eligibility for potential life-prolonging precision therapies. Tumor genetic testing is potentially not necessary for men with only localized PCa.

In this second part, we highlight how genetic testing can lead to improved, life-prolonging mCRPC therapeutic strategies based on a review of the recent phase III trials and subsequent regulatory approvals for PARP inhibitors in mCRPC.

### Implications of genetic testing results on clinical outcomes – the devil is in the details

Based on the comparison of primary prostate tumors and subsequent metastases at the time of castration resistance, DNA damage repair (DDR) mutations appear to occur *early* in the evolution of most cases of advanced PCa [11–13]. These mutations have profound implications for a patient’s course and response to therapies.

However, there are many questions regarding genetic testing results that are often overlooked by the broader community. These include:

1. *Does the presence of a BRCA versus non-BRCA HRR mutation impact treatment outcomes differently?*
2. *Does the origin of a mutation (i.e. somatic vs. germline) affect outcomes?*
3. *Does it matter whether one (monoallelic) or two (biallelic) alleles for a DDR gene are mutated?*
4. *Does the nature of that mutation (e.g. deletions versus other types) affect treatment response?*
5. *How do co-occurring additional genetic alterations impact response and resistance to treatment?*

Such variables create a myriad of potential testing outcomes. However, patient numbers for any given testing outcome are often limited. Therefore, one must exercise caution when drawing conclusions for how any specific mutation signature may affect long-term outcomes.

First we will review the therapeutic implications of each issue in the context of the natural history of PCa and response to more conventional PCa therapies (i.e. androgen receptor signaling inhibitors, ARSIs/taxanes) [14]. We will then explore these issues in the context of PARP inhibitors.

### HRR mutations associated with inferior outcomes

#### *BRCA* versus non-*BRCA* mutations

Numerous studies have documented that *BRCA2-*disrupted tumors represent a unique and clinically relevant molecular subtype of aggressive PCa [15]. Given the aggressiveness and genomic complexity/heterogeneity of *BRCA2*-mutated disease, one may expect the presence of a *BRCA2* mutation to lead to worse patient outcomes [3, 16, 17].

Recent next-generation-sequencing (NGS) studies have revealed that pathogenic germline mutations in DDR genes are present in 8%-12% of patients with metastatic PCa [18]. The most frequently mutated gene is *BRCA2* (~5–6%), with *BRCA1* and other DDR genes (e.g. *Partner and localizer of BRCA2 (PALB2), Checkpoint kinase 2 (CHEK2), Ataxia-telangiectasia-mutated (ATM)*each occurring in <1% of patients.

PROREPAIR-B [18] was the first prospective study to estimate how *germline* homologous recombination repair (HRR) alterations impact PCa-specific survival (PCSS). PCSS was halved in patients who were germline *BRCA2* carriers compared with noncarriers (17.4 vs. 33.2 months *P* = *0.027*). By contrast, there was not a significant difference in PCSS comparing *ATM/BRCA1/CHEK2/PALB2*carriers to noncarriers (23.3 versus 33.2 months, *P* = *0.264*). Germline *BRCA2* mutation is a particularly poor marker for patient outcomes.

A recent multicenter observational study of 729 PCa patients (“CAPTURE”) compared the outcomes of patients both with/without somatic and germline HRR mutations [14]. When comparing three different groups – *BRCA*-mutated vs. HRR-mutated-non-*BRCA* vs. non-HRR mutated – the radiographic progression-free survivals (rPFSs) were 7.1 vs. 9.0 vs. 10.7 months, with all differences significant. Similar significant trends were found in overall survival (OS) (18.4 vs. 21.9 vs. 29.1 months, respectively). These data reiterate the inferior outcomes of patients with BRCA mutations.

#### Somatic versus germline mutations

While patients with germline *BRCA2* mutations are known to have a more aggressive disease course [13, 14, 19–21], most reports of HRR alterations in PCa comparing the prognostic implications of somatic versus germline alterations have been somewhat inconclusive. Many studies do not draw conclusions about how mutation origin impacts patient outcomes.

In CAPTURE, somatic mutations were more common than germline mutations in HRR genes overall (23.2% versus 7.4%). In both the BRCA cohort and the HRR-mutated non-*BRCA* cohort, somatic mutations outnumbered non-somatic mutations by ~3 to 1. Similarly, in the non-*BRCA*, HRR mutated cohort, somatic-only mutations outnumbered germline mutations (13.4% vs. 4.0% respectively). Of note, somatic *BRCA1/2* mutations were significantly more common than germline *BRCA1/2* mutations (9.8% vs. 3.4% respectively) [22]. Interestingly, patients with alterations in *BRCA1/2* were more likely to have co-occurring non-HRR somatic or germline mutations (28.1%) compared to patients with other gene alterations (10.2%), likely indicative of genomic instability.

In sum, this data suggests the origin of a mutation (i.e. somatic tumor tissue versus germline) may does not have a major impact on its prognostic significance in metastatic prostate cancer.

#### Patients with germline BRCA2 vs. BRCA 1 mutations

Important differences exist between *BRCA2* and *BRCA1* mutated patients. In one study the prevalence of germline *BRCA2* mutations was nearly 5x the rate of *BRCA 1* (5.3 vs. 0.9% respectively) [23]. In the largest pan-cancer analysis of BRCA1/2 alterations published to date [24] (including 7186 prostate cancers), it was shown that germline mutations were present in 35% of patients with *BRCA1*-altered mCRPC compared with 50% of patients with *BRCA2* altered mCRPC.

#### HRR gene zygosity

Two (biallelic) or one (biallelic) loss of function (LOF) alterations are possible for a given DDR gene. These alterations can come in many forms including allelic loss/deletion, frameshift insertions/deletions, and deleterious missense or truncating mutations. In the context of a pathogenic germline mutation impacting one allele, the tumor generally acquires a second oncogenic somatic ‘hit’ in the wild-type allele typically through deletion of the wild-type allele or duplication of the mutation, a process called loss of heterozygosity (LOH) which results in biallelic loss and presumed DNA repair deficiency. *BRCA1* mutations are more likely to be bystander mutations in the context of high tumor mutational burden (TMB)/Microsatellite-high (MSI-high) disease and did not lead to HRR deficiency and as a result were more resistant to PARP inhibition [22, 25]. By contrast, *BRCA2* mutations are more likely biallelic, leading to loss of function in HRR, and more responsiveness to PARP inhibition [25].

Zygosity is highly dependent on tumor purity and difficult to discern. The most careful studies found an 80% rate of biallelic BRCA inactivation in PCa [26]. Thus drawing conclusions based on zygosity is difficult. This is further complicated by the fact that most multi-institutional studies rely on the provision of archival tissue and most commercial NGS testing does not report zygosity or loss of heterozygosity.

Despite these limitations, in CAPTURE, biallelic mutations (14.7%) were approximately equal in prevalence to monoallelic mutations (15.9%). While biallelic alterations were slightly more frequent than monoallelic in the BRCA subgroup (56% versus 44% respectively), in the non-BRCA subgroup most patients presented with monoallelic mutations (58.3%). Despite some small differences in PFS and OS, in no study was there a significant difference observed for those with biallelic versus monoallelic alterations [14]. This suggests, with extreme caution about the interpretation of the results, that the zygosity of an HRR mutation may not have a major impact on responses to ARSIs/taxanes. However, the lack of difference in this study and others may be a function of inadequate identification of zygosity and testing artifacts rather than a real biological finding.

#### Prospective Clinical Trials

Clinical trials have confirmed that *BRCA-*mutated tumors have worse outcomes. Exploratory analyses of the control arms (i.e. ARSI only arms) of the three phase III trials evaluating the benefit of first-line ARSIs combined with PARP inhibitors (PROPEL [2], MAGNITUDE [4] and TALAPRO-2) [27] demonstrated that patients with BRCA-mutated tumors had a lower rPFS and OS than patients with non-BRCA HRR mutated and non-HRR mutated tumors. In addition, patients with *all* HRR-mutations, including *BRCA* patients, had worse responses to ARSI-monotherapy than non-HRR tumors [2, 4, 27].

In a study of 319 patients in which 7.5% were found to be DDR deficient, patients with germline DDR defects exhibited attenuated responses to AR-targeted therapy, with a median PFS of 11.8 months after starting ADT and a median time to PSA progression on first-line AR targeted therapy of 3.3 months (95% CI 2.7–3.9 months) [28]. Importantly, whether the mutations were germline/somatic or mono- versus biallelic *did not* alter these conclusions. Of note, patients with mutations outside of *BRCA2* had similar treatment responses as patients without any HRR mutations [29, 30].

#### Therapeutic Sequencing

Studies have also tried to determine the optimal sequence of ARSIs and taxanes in the mCRPC treatment paradigm for HRR mutations. In PROREPAIR-B, PCSS for germline *BRCA2* carriers was superior for those treated first with ARSIs followed by taxanes (*P* = 0.014) than the reverse. Of note, for non-HRR germline patients, both sequences were equivocal.

These data imply that optimal first-line mCRPC treatment options may be heavily informed by genetic testing, particularly for patients with *BRCA2* mutations. Thus, not only do germline *BRCA2* mutations have a detrimental impact on mCRPC outcomes, but *BRCA2* mutations may benefit from exposure first to ARSIs followed by taxanes.

#### Summary of Implications for ARSIs and taxanes

Overall, patients with BRCA mutations had *significantly* worse treatment outcomes than non-*BRCA* HRR mutated or non-HRR tumors, *regardless* of whether mutations present were somatic or germline alterations. Suboptimal responses to ARSIs or taxanes may be a clue that a *BRCA* mutation (specifically *BRCA2*) is present and may prompt genetic testing.

### PARP inhibitors in mCRPC

PARP inhibitors are the only agents that have a demonstrated survival benefit *specifically* for patients with HRR mutations, particularly *BRCA2* mutated carriers. It is critical to identify these patients early so that PARP inhibitors can be made available to the patient.

#### Evidence for PARP inhibitors in later stage mCRPC

Multiple phase III clinical trials have demonstrated the efficacy of PARP inhibitor monotherapy in patients with mCRPC with HRR mutations after progression on ARSIs (Table 1). Rucaparib is FDA-approved to treat patients with mCRPC with deleterious germline or somatic *BRCA1* or *BRCA2* mutations and who had previously received an ARSI and taxane-based chemotherapy. Olaparib is FDA-approved for the treatment of mCRPC in a broader panel of 1 of 14 deleterious germline or somatic mutations in HRR genes after previously receiving an ARSI.

**Table 1:** Completed trials of PARPi monotherapy in mCRPC.

Clinical Trial	NCT#	PARPi Studied	Phase	HRR Selection Status	Prior line(s) of therapy required	Experimental Arm	Control Arm	Outcome of Primary Endpoint	Reference
TOPARP-A	01682772	Olaparib	II	Unselected	ARSI, chemotherapy	N.A. Single Arm		88% composite RR in HRR+	[55]
TOPARP-B	01682772	Olaparib	II	HRR+	ARSI, chemotherapy	N.A. (2 dosing cohorts)	N.A. (2 dosing cohorts)	400 mg dose cohort: 54% composite RR 300 mg dose cohort: 39% composite RR	[38]
PROfound	02987543	Olaparib	III	Cohort A: BRCA1/2, ATM Cohort B: other HRR+	ARSI	Olaparib	Physician’s choice of enzalutamide or abiraterone	Cohort A: rPFS improved 7.4 months (olaparib) vs. 3.6 months (ARSI), HR 0.34	[31]
TRITON2	02952534	Rucaparib	II	BRCA1/2	ARSI x2, chemotherapy	N.A. Single Arm		ORR 43.5%	[35, 56]
GALAHAD	02854436	Niraparib	II	HRR+	ARSI, chemotherapy	N.A. Single Arm		BRCA1/2: ORR 34.2% Non-BRCA: ORR 10.6%	[36]
TALAPRO-1	03148795	Talazoparib	II	HRR+	ARSI, chemotherapy	N.A. Single Arm		ORR 29.8%	[57]

Adapted from Taylor et al., Frontiers in Oncology, 2020. *NCT* clinicaltrials.gov registration, *ARSI* Androgen receptor signaling inhibitor, *HRR* homologous recombination repair, *HRR+* homologous recombination repair deficient, *RR* response rate, *ORR* overall response rate, *rPFS* radiographic progression-free survival, *HR* hazard ratio.

In the phase III PROfound trial [1, 31] olaparib was associated with longer progression-free survival (PFS) and improved clinical responses in patients with mCRPC who previously progressed on enzalutamide or abiraterone and had an HRR gene alteration, when compared to physician’s choice of enzalutamide or abiraterone. Patients were required to have somatic or germline HRR gene mutations and were allocated to one of two cohorts: Cohort A with *BRCA1/2* or *ATM* mutations and Cohort B comprised of patients with a mutation in at least one of 12 other prespecified HRR genes.

At the time of the trial’s initial reporting in the entire population (cohorts A + B), rPFS was significantly longer in the olaparib arm (HR 0.49, 95% confidence interval (CI) 0.38–0.63, *P* < 0.001). While OS was also significantly improved with olaparib (HR 0.69, 95% CI 0.50–0.97, *P* = 0.02), it was only statistically significant for cohort A, but trended towards improved mOS in the overall population (HR 0.79, 95% CI 0.61–1.03) [1]. Of note, superior clinical benefit with olaparib versus control was found regardless of whether patients previously received taxanes, and the benefits were largely observed in the BRCAm subgroup, not the ATMm subgroup [32].

At the final analysis, updated PROfound data demonstrated that olaparib was associated with a significantly longer rPFS (HR 0.22) and OS (HR 0.63) than the control group [32]. This was particularly true for patients with *BRCA2* homozygous deletions who experienced prolonged responses to olaparib (*n* = 16, median rPFS 16.6 months), with similar responses in both germline or somatic mutation [32].

TRITON3 found similar benefits when the PARP inhibitor rucaparib was compared to physician’s choice of chemotherapy or second-generation ARSI in chemotherapy naïve mCRPC patients with *BRCA1/2 or ATM* alterations. Radiographic PFS was significantly improved with rucaparib (HR 0.61, HR 0.47–0.80, *P* < 0.001), with a trend towards improved OS, but again largely observed in patients with mCRPC and BRCAm rather than ATMm [33, 34]. This data supports the use of a PARPi prior to a taxane in BRCAm mCRPC.

The NCCN guidelines recommend olaparib for patients with HRR-mutated mCRPC. Similarly, NCCN recommends rucaparib for patients with *BRCA* mutated mCRPC who have progressed on prior novel hormone therapy and/or chemotherapy. However, only olaparib has been shown to improve survival in a phase 3 trial and is thus the preferred PARPi for monotherapy use.

#### BRCA2 patients are most responsive to PARP inhibition

The specific HRR gene that is mutated impacts response to PARP inhibition. Efficacy in PROfound was primarily driven by patients with *BRCA* mutations, specifically *BRCA2*. *BRCA2’s* prevalence dramatically outweighs *BRCA1* (90.6% versus 9.4% respectively of *BRCA* mutations). Patients with *BRCA2* mutations experienced deep rPFS benefits (HR 0.22, 95% CI 0.15–0.32, *P* < 0.001) and OS benefits from olaparib (HR 0.59, 95% CI 0.37–0.95) whereas the HR for OS in all patients was not statistically significant (HR 0.79, 95% CI 0.61–1.03) [32]. The survival benefit was also not statistically significant in *ATM* patients. The small number of patients with mutations other than *BRCA1/2* or *ATM* limits the trials’ ability to draw conclusions about these groups.

PARP inhibitor efficacy is diminished in *BRCA1* versus *BRCA2* altered mCRPC [22, 25]. One study pooled the efficacy data for a range of PARP inhibitors for mCRPC and compared the outcomes in those with deleterious *BRCA1* versus *BRCA2* mutations. Rucaparib’s activity seemed to be generally greater in patients with *BRCA2* alterations than patients with *BRCA1* alterations, as evidenced by PSA_50_ (60% versus 15% respectively), ORR (45% versus 33%) and median rPFS (9.7 vs. 8.7 months). This apparent discrepancy in PARP inhibitor sensitivity between patients with *BRCA2* and *BRCA1* mutated disease is not restricted to rucaparib but is a class effect of all PARP inhibitors.

This observed difference has been hypothesized to be explained by several factors:

- ***BRCA2*** **alterations are more likely to be biallelic than** ***BRCA1*:** In TRITON2, only 40% of *BRCA1* mutations that were evaluable for zygosity status were biallelic alterations, whereas 85% of evaluable *BRCA2* alterations were biallelic [35]. In a large pan-cancer *BRCA1/2* analysis, biallelic inactivation occurred in only 50% of *BRCA1* mutants versus 90% of *BRCA2* mutants. Therefore, biallelic mutations in PCa seem to involve *BRCA2* more than *BRCA1*. *BRCA1* mutations are more likely to be passenger events in the setting of MSI-H, TMB-high disease [22, 25].
- ***BRCA1*** **mutated cancers have more genomic co-alterations (e.g. in** ***TP53*** **or** ***PTEN*) than** ***BRCA2***: TP53 alterations portend a worse prognosis in most malignancies. In TRITON2, patients with *BRCA1* mutations had more frequent co-alterations in TP53 (62%) and PTEN (69%) compared with those who had *BRCA2* mutations (42 and 29% respectively). Deleterious TP53 alterations were significantly more frequent in *BRCA1* mutated versus *BRCA2* mutated PCa.

#### Sensitivity to PARP inhibitors for non-BRCA HRR Mutants

PARP inhibition is less effective in patients with non-BRCA HRR gene mutations (Table 1). The non-*BRCA* non-*ATM* cohort in PROFOUND had neither a significant rPFS benefit nor OS benefit from olaparib compared with control [1, 31]. Similar non-significant results for non-*BRCA* patients were seen in other PARP inhibitor monotherapy trials, including TRITON-3 (rucapaib), GALAHAD (niraparib), TALAPRO-1 (talazoparib), and TOPARP-B (olaparib) [33, 36–38]. Similarly, patients with *CDK12* mutations have also been found to have aggressive disease, with high rates of metastases and shortened OS [39, 40]. These patients have also been shown to not respond well to hormonal therapy, PARP inhibitors, or taxanes. However, the therapeutic implications of *CDK12* mutations are still an active area of investigation [22, 24, 25].

Outside of some cases of sensitivity in *PALB2* and *RAD51* mutations, these trials all found very low response rates, short PFS and low PSA declines from PARP inhibitor monotherapy in non-*BRCA2-mutated patients*, including *ATM*, *CHEK2*, and *CDK12*. Expectations for PARP inhibitors are tempered for patients with such mutations.

In addition, there may be other mutations not currently identified in the canonical HRR gene sets. These include *RNASEH2* loss *MMS22L* loss, and others [41, 42]. These genes and their therapeutic implications are active areas of investigation.

#### Effect of other variables on PARP inhibitor monotherapy efficacy

TRITON2 examined the activity of rucaparib according to genetic origin (germline or somatic), zygosity status (monoallelic or biallelic) and mutation type (homozygous deletion or other deleterious mutation). The efficacy of rucaparib was generally greater (although not always statistically superior) in patients with germline compared with somatic *BRCA1/2* mutations (PSA_50_ response of 61% versus 51% with similar ORR estimates). Efficacy was also higher in biallelic versus monoallelic mutations (PSA50 response 75% versus 11% respectively; ORR 52% vs. 50%) and in patients with homozygous deletions versus other deleterious mutations (PSA_50_ response 81% versus 53%; ORR 60% versus 38%). Some of these trends, albeit non-significant, were also born out in TRITON3 [33].

Similarly, a recent post hoc analysis of PROfound attempted to determine whether these same issues affected efficacy outcomes. Olaparib was associated with prolonged rPFS and higher confirmed ORR for patients with either germline (rPFS HR 0.08) or somatic (HR 0.16) *BRCA* alterations compared with control. The numbers of evaluable patients were underpowered to make OS comparisons. Of note, similar results have been reported in the TOPARP-B study [38] of olaparib, and the rucaparib studies TRITON2 and TRITON3 [33, 35, 43]. Hence, although germline status identification is relevant for estimating patient and relatives’ cancer risk, whether a mutation was germline or somatic was not found to materially impact responses to olaparib in PROfound.

Of note, in PROfound patients with homozygous *BRCA2* deletions (*n* = 16), which may include those that have both one germline and then a second-hit somatic mutation, experienced a significantly prolonged response to olaparib (16.6 m) compared to those with *BRCA1* and/or *ATM* gene mutations. This is consistent with observations from TOPARP-B. This result suggests exceptional responses to PARP inhibition may be seen in patients with “two hit” events (inheriting a germline *BRCA2* alteration and then acquiring a second somatic *BRCA2* alteration later or possessing two aberrant somatic alleles). Similarly, patients with deletions, where secondary BRCA2 reversion mutations cannot emerge because of complete gene absence, may be exceptional responders to PARP inhibition [17, 44, 45].

However, response to olaparib was not restricted to biallelic BRCA mutations. While the ORR in patients with biallelic BRCA mutations was 60.7%, ORR in the heterozygous group (i.e. no evidence of a second hit loss) was still 44.4%. Tumor responses were also observed among the small number of patients with heterozygous loss and those where zygosity could not be determined (36% of cases). This data supports the idea that, regardless of zygosity, identification of any pathogenic BRCA alteration, particularly *BRCA2*, is sufficient to identify patients who may benefit from PARP inhibition – even without clear evidence of second HRR mutation inactivation events. These results are consistent with previous studies of BRCA alterations in other BRCA-driven tumor types, regardless of whether patients still benefited from PARP inhibitors regardless of whether BRCA *alterations* were biallelic or heterozygous “one hit” events [26, 35, 46].

A similar observation was made in TRITON-2 for individuals with *both* germline and somatic HRR mutations (so-called “double hit” patients). TRITON-2 evaluated rucaparib monotherapy in later-stage mCRPC [35]. While zygosity was only determined in ~40% of patients, the rates of PSA response were found to be slightly higher in the biallelic subgroup.

#### First-line combination of PARP inhibitors and ARSIs in mCRPC

Preclinical investigations suggested potential synergy when co-targeting the androgen receptor and DNA repair mechanisms [47, 48]. ARSIs were shown to down-regulate DNA repair genes, enhancing HRR deficiency. This led to several phase III clinical trials investigating the first-line combination of ARSI (enzalutamide or abiraterone) with a PARP inhibitor (olaparib, talazoparib, or niraparib) which have recently reported data. Combination therapy was found to have a PFS advantage relative to ARSI alone (Table 2), suggesting a synergistic antitumor effect and moving PARP inhibition to first-line therapy.

**Table 2:** Completed first line trials of PARP inhibitors combined with ARSI in mCRPC.

Combination Class	Trial, NCT#	PARPi	Combined Agent	Phase	HRR Selection Status	Primary Outcome(s)	Primary Outcome in BRCA Mutated Patients	Primary Outcome in All HRR Mutated	Reference
Androgen Signaling Inhibitor (ARSI) therapy	TALAPRO-2 (NCT03395197)	Talazoparib	Enzalutamide	III	Unselected	Median rPFS Not reached HR 0.63, *p* < 0.001 (1) Median Follow-up for rPFS was 24.9 and 24.6 months for experimental and control arms respectively	HR 0.20, *p* < 0.0001	Median rPFS was not reached. HRR-deficient subgroup HR = 0.46 (95% CI 0.30–0.70, *p* < 0.001). Median follow-up for rPFS was 17.5 months and 16.8 months for the talazoparib and placebo groups, respectively	[3, 58]
	BRCAAway (NCT03012321)	Olaparib	Abiraterone	II	Inactivating BRCA1/2 and/or ATM Alterations	Median rPFS HR 0.28 *P* < 0.001 (2)	HR 0.28, *p* = 0.07	N.A.	[59]
	{NCT01972217}	Olaparib	Abiraterone	II	Unselected	Median rPFS 13.8 mo vs. 8.2 mo HR 0.65	N.A.	Median rPFS 17.8 mo vs. 6.5 mo HR 0.74, *p* = 0.58	[16]
	PROpel (NCT03732820)	Olaparib	Abiraterone	III	Unselected	Median rPFS 24.8 mo vs. 16.6 mo HR 0.66	N.A.	HR 0.5 (95% CI 0.34–0.73)	[2]
	MAGNITUDE (NCT03748641)	Niraparib	Abiraterone	III	A: Unselected B: HRR+	A: No improvement in rPFS, HR 1.09 B: Improved rPFS 16.5 mo vs. 13.7mo HR 0.73	Median PFS 19.5 vs. 10.9 HR 0.55 *P* = *−*0.0007	Median PFS 16.7 mo vs. 13.7 mo HR 0.76 (95% CI 0.69–9.97, *P* = 0*.*0280	[4]
	NCI 9102 (NCT01576172)	Veliparib	Abiraterone	II	Unselected	PSA_50_ 72% vs. 64%	N.A.	N.A.	[60]

NCT clinicaltrials.gov registration, *ARSI* Androgen receptor signaling inhibitor, *HRR* homologous recombination repair, *HRR+* homologous recombination repair deficient, *RR* response rate, *ORR* overall response rate, *rPFS* radiographic progression-free survival, *HR* hazard ratio.

(1) For TALAPRO-2 data, this refers to the talazoparib+enzalutamide arm in all comers (Cohort 1).

(2) For BRCAAway, data shown compares the combination arm to the abiraterone/prednisone monotherapy arm. Olaparib monotherapy arm not shown.

A recent meta-analysis of PROpel, MAGNITUDE, and TALAPRO-2 compared rPFS, OS, and adverse events between the treatment arms [49]. The addition of PARP inhibition to ARSI in first-line mCRPC was associated with rPFS benefits across subgroups, with the greatest magnitude of benefits with BRCA1/2. OS benefits remained inconsistent, irrespective of HRR mutation status, with significant increases in grade 3 or higher treatment-related adverse events, particularly anemia. This meta-analysis suggests this combined approach should be selectively offered to patients with HRR mutations, preferentially *BRCA1/2*.

Below is a brief discussion of the clinical trial evidence that led to the FDA approvals of several first-line mCRPC options combining ARSIs and PARP inhibitors. The NCCN guidelines recommend first-line olaparib plus abiraterone and niraparib plus abiraterone for patients with *BRCA* mutations and talazoparib+enzalutamide for patients with any alteration in a specified HRR gene panel.

### Abiraterone plus olaparib

PROpel was the first positive phase 3 trial of ARSI + PARP inhibitor. PROpel evaluated olaparib ± abiraterone in a biomarker unselected first-line mCRPC population and retrospectively sought to assign HRR status. While the study met its primary endpoint showing a statistically significant and clinically meaningful rPFS benefit from olaparib+abiraterone versus the abiraterone monotherapy arm (24.8 months versus 16.6 months respectively, HR 0.66, 95% CI 0.54–0.81, *P* < 0.001) the benefit was primarily driven by *BRCA2* mutations (median rPFS not reached in experimental arm compared with 8.4 months in placebo arm; *BRCA2* represented 12% of patients).

For patients with HRR mutations, the median OS was 42.1 months in the olaparib+abiraterone arm versus 34.7 months for the abiraterone monotherapy arm (maturity 47.9%, HR 0.81, CI 0.67–1.00, *P* = 0.0544). While OS trended towards a benefit in the olaparib+abiraterone arm in the overall HRR group, the benefit was largest in the *BRCA2* mutated group comparing the combination and placebo arms (Not reached versus 23.6 months, HR 0.29, 95% CI 0.14–0.55, *P* < 0.001) [50]. OS results were non-significant in the non-HRR mutated, and non-*BRCA* mutated subgroups.

These findings led to the FDA approval of first-line olaparib with abiraterone for the treatment of pathogenic *BRCA1/2* mutated mCRPC, now adopted by numerous society guidelines [50]. In Europe, this combination was approved in the first-line mCRPC setting for patients not requiring chemotherapy, irrespective of HRR status, as per the study design.

To parse the effects from each individual therapy, BRCAAway was a recent phase II trial that randomized first line mCRPC patients with inactivating *BRCA1,2* and/or *ATM* alterations 1:1:1 to abiraterone monotherapy, olaparib monotherapy, and olaparib plus abiraterone. Combination therapy showed a PFS benefit compared against both abiraterone (HR 0.28, 95% CI 0.13–0.65) and olaparib (HR 0.32, 95% CI 0.14–0.75) monotherapies. Median PFS comparing the abiraterone monotherapy vs. olaparib monotherapy versus combination arm were 8.4 months vs. 14 months vs. 39 months respectively. The median PFS from the combination supports a synergistic effect for the combination of ARSI with PARP inhibition.

### Enzalutamide plus talazoparib

TALAPRO-2 compared enzalutamide±talazoparib in untreated mCRPC. Similar to PROpel, there was an rPFS benefit in all patients. Median rPFS was not reached for the talazoparib+enzalutamide arm and 21.9 months for the placebo+enzalutamide arm (HR 0.63, 95% CI 0.51–0.78, *P* < 0.0001), the benefits were driven mostly by patients with HRR gene deficiencies (HR 0.46, 95% CI 0.30–0.70, *P* = 0.0003). Again, the largest benefits were seen in *BRCA1/2* patients (HR 0.23, 95% CI 0.10–0.53, *P* = 0.0002). OS data for TALAPRO-2 is still immature. Based on these results, the FDA approved talazoparib+enzalutamide for patients with HRR-mutated mCRPC. Similar to abiraterone plus olaparib, in Europe talazoparib+enzalutamide is approved for all adult mCRPC patients in whom chemotherapy is not clinically indicated, regardless of HRR mutation status.

### Abiraterone plus niraparib

Another PARP inhibitor, niraparib, was studied in first-line combination with abiraterone in the phase 3 trial MAGNITUDE [4]. After a median of 18.6 months, rPFS was improved for in the niraparib arm for patients with HRR mutations (HR 0.73, 95% CI 0.56–0.96, *P* = *0.022*), again driven mainly by the *BRCA* subgroup (HR 0.53, 95% CI 0.36–0.79, *P* = *0.001*). rPFS was ***not*** shown to be improved in non-BRCA HRR mutations, and futility was declared in patients with non-HRR mutations. OS benefit from combination therapy was also seen (HR 0.54, 95% CI 0.33–0.90, nominal *P* = 0.0181). Based on these results, the FDA approved niraparib+abiraterone for BRCA-mutated mCRPC patients. However, the lower efficacy in all subsets of patients (BRCA, HRR, and non-HRR) and higher degree of toxicity such as anemia limited the enthusiasm for this combination as compared to abiraterone+olaparib or enzalutamide+talazoparib.

## Population studies further scrutinize clinical trial outcomes

The existing clinical trials for PARP inhibitors are limited by the small number of patients with specific HRR mutations available for study. A recent pooled analysis of multiple trials of PARP inhibitors in mCRPC investigated the efficacy of PARP inhibitors on each individual HRR gene [51]. Of note, this study included both PARP inhibitor+ARSI first-line trials and PARP inhibitor monotherapy second-line trials.

In this pooled analysis, once again the benefit from PARP inhibitors was greatest for *patients with altered BRCA2* (*n* = 422, rPFS HR 0.31, OS HR 0.66), followed by *BRCA1* (*n* = 64, rPFS HR 0.51, OS HR 0.74, neither significant). Benefits were also seen in patients with *CDK12* (median rPFS HR 0.50, OS HR 0.63, *P* < 0.01) and *PALB2* (rPFS HR 0.52, OS HR 0.78) mutated patients. Of note this study did *not* find a significant benefit in patients with *CHEK2* or *ATM* mutations. While these studies need to be interpreted with caution given the small sample sizes, they reiterate the concern that the benefits from PARP inhibitors may only be seen in a small subset of patients with HRR mutations.

## Immunotherapy and genetic testing

Durable efficacy from pembrolizumab has been observed in patients with microsatellite-instability high (MSI-H) mCRPC [52]. Between 1–3% of mCRPC patients have been identified as MSI-H through circulating tumor DNA (ctDNA) alone [53]. Somatic and germline alterations in microsatellite stability, tumor mutational burden (TMB) may help identify patients who might receive durable clinical benefits from immunotherapy [54]. While high-TMB, microsatellite stable (MSS) patients are uncommon, there is some suggestion of ICI benefit in this population.

## Conclusions

The introduction of PARP inhibitors has revolutionized PCa treatment. These therapeutic advances reiterate the recommendation that all metastatic PCa patients should now undergo both germline and somatic testing at time of diagnosis. We also reiterate our recommendation for germline testing for all patients with PCa. In addition, serial ctDNA testing may detect novel actionable HRR mutations or MSI-high disease. Improved genetic testing techniques combined with improved knowledge of therapeutic will continue to advance precision medicine in PCa.
